# Gamma delta T cells in non-immune patients during primary schistosomal infection

**DOI:** 10.1002/iid3.18

**Published:** 2014-05-16

**Authors:** Eli Schwartz, Etti Rosenthal, Ilan Bank

**Affiliations:** 1 The Center for Geographical Medicine & Tropical Diseases, Chaim Sheba Medical CenterTel Hashomer, 52621, Israel; 2 Institute of Hematology, Chaim Sheba Medical CenterRamat Gan, Israel; 3 Department of Medicine F and Laboratory for Immunoregulation, Chaim Sheba Medical CenterRamat Gan, Israel

**Keywords:** Acute schistosomiasis, gamma delta T cells, S. *hematobium*, *S. mansoni*, travelers

## Abstract

The mevalonate pathway is critical for the survival of *Schistosoma*. γδ T cells, a small subset of peripheral blood (PB) T cells, recognize low molecular weight phosphorylated antigens in the mevalonate pathway, which drive their expansion to exert protective and immunoregulatory effects. To evaluate their role in schistosomiasis, we measured γδ T cells in the PB of non-immune travelers who contracted *Schistosoma hematobium* or *Schistosoma mansoni* in Africa. The maximal level of γδ T-cells following infection was 5.78 ± 2.19% of the total T cells, versus 3.72 ± 3.15% in 16 healthy controls [*P* = 0.09] with no difference between *S. hematobium* and *S. mansoni* in this regard. However, among the nine patients in the cohort who presented with acute schistosomiasis syndrome (AS), the level (3.5 ± 1.9%) was significantly lower than in those who did not (8.6 ± 6.4%, *P* < 0.05), both before and after therapy. Furthermore, γδ T cells increased significantly in response to praziquantel therapy. In a patient with marked expansion of γδ T cells, most expressed the Vδ2 gene segment, a hallmark of cells responding to cognate antigens in the mevalonate pathways of the parasite or the human host. These results suggest an immunoregulatory role of antigen responsive γδ T cells in the clinical manifestations of early schistosomal infection.

## Introduction

Schistosomiasis is a helminthic infection caused by blood-flukes of the genus *Schistosoma*. The main species causing human disease are *S. mansoni*, and *S. haematobium* (in Africa) and *S. japonicum* (in Southeast Asia). Approximately 230 million people are infected with *Schistosoma* species in at least 76 countries, mainly in Africa [1]. The increasing number of travelers from industrialized to developing countries has resulted in acquisition of the disease by non-immune travelers from non-endemic areas as well. For example, in Israel, a country free of endogenous schistosomiasis, infection of young Israeli travelers to Africa (where the two dominant species are *S. hematobium* and *S. mansoni*), has resulted in a significant number of imported cases [2].

The initial type 1 helper T cell (T_H_1)-response to the acute schistosomal infection targets adult parasites, but typically transitions to a T_H_2-type response after the parasite's eggs are produced [3]. Perioval granulomatous inflammation is mediated by antigen specific CD4^+^ T cells. The typically prevalent T_H_2-type reaction is associated with mild lesions consisting of small granulomas comprised of eosinophils, macrophages, and lymphocytes with an increasingly fibrotic extracellular matrix. Tissue fibrosis, stimulated by interleukin (IL)-13 and other cytokines, can become pathological, a detrimental effect of chronic T_H_2-type responses. The immune response to *Schistosoma* derived peptides is primarily mediated by activated CD4^+^ T cell receptor (TCR) αβ cells [4–6]. IL-4, 5, and 13 secreted by these cells, lead to eosinophilia, and help B cells to produce *Schistosoma*-specific antibodies, which are hallmarks of primary schistosomal infection [7].

Another subset of lymphocytes, γδ T cells, are CD4-CD8- CD3+ T cells that express a TCR encoded by the γ and δ genes, and consist about ∼5% of the circulating PB T cells [8]. These cells are known to participate in the immune response against infectious organisms, and numerical perturbations of γδ T cells exceeding 30% of the PB T cells in some cases, have been documented in a variety of infectious diseases [9]. In particular γδ T cells that express a TCR encoded by the Variable (*V*) γ9 and δ2 genes recognize powerful microbial antigens, primarily (*E*)-4-hydroxy-3-methyl-but-2-enyl pyrophosphate (HMBPP) produced in the alternative mevalonate pathway of some parasites and bacteria, and secrete primarily T1 type cytokines in response [10,11]. Isopentenyl pyrophosphate (IPP) is another albeit, less powerful, antigen produced in the mevalonate pathway of both eukaryotes and prokaryotes [11]. While γδ T cells have been shown to be recruited to egg-induced granulomata during infection of experimental animals [12,13] evidence for involvement of γδ T cells in human schistosomiasis is very limited. The one publication broaching this subject reported that γδ T-cells are expanded in the PB of patients with bladder cancer related to chronic schistosomiasis relative to bladder cancer patients without schistosomiasis [14].

Because the mevalonate pathway appears to play a critical role in the survival of *Schistosoma*, and has even been proposed as a therapeutic target, the γδ T cell response to human schistosomiasis is of great potential interest [15]. Thus, the goal of this study was to evaluate γδ T-cells in PB of previously healthy non-immune individuals with primary schistosomal infection, and to correlate the level of these cells in the PB with the clinical syndromes developing in the patients.

## Materials and Methods

### Patients

The 18 patients included in the study were non-immune travelers who contracted infections with either *S. mansoni or S. hematobium* for the first time during their travel in Africa in an area endemic for schistosomiasis. All patients were seen in the Center for Geographical Medicine at the Sheba Medical Center upon returning from their trip to endemic areas after developing symptoms of AS. The asymptomatic patients were companions to the same exposure of the symptomatic patients who came for serology screening to assess whether they too were infected.

Diagnosis was made by detecting eggs in the urine or stool, and/or by serology performed at the division of parasitic diseases at the Center for Disease Control (Atlanta, USA). All sera were initially screened by the FAST-enzyme linked immunosorbent assay (ELISA), positive sera were considered those registering >8 units and they were confirmed by immunotransfer blot. The serology test is highly sensitive (99%) and specific (99%) [16,17]. All patients in the study tested positively for antibodies by this methodology.

Treatment consisted of a course of praziquantel (60 mg/kg in two divided doses). Controls were healthy people matched for age and sex. Blood samples were drawn for evaluations of complete blood count (CBC), schistosomal serology, and T-cell antigens upon the first clinic visit, and for 10 patients, 2–4 weeks after treatment (one patient had evaluation only after treatment). Immunological study of γδ T cells was approved by the Helsinki committee of the Sheba Medical Center. The other tests of individual patients was carried out as and when they presented and independently of the others.

### Determination of lympocyte subsets

Peripheral blood mononuclear cells (PBMC) were stained with monoclonal antibodie*s* (mAb) TCR1 [directed against constant region of TCRγ chain (Cγ)], that identifies all γδ T cells, or with mAb to Vδ1 or Vδ2 in individual instances (Fig. 3), to identify mutually exclusive γδ T cell subsets expressing the Vδ1 or Vδ2 gene segments. Percent of total γδ cells, or of the Vδ1 or Vδ2 subsets within the total T cell population within the gated lymphocyte (L) population (above background stain with isotype control mAb) was recorded using the installed computer software. To obtain percent of γδ T cells of all CD3+ T cells, the percent of those staining with mAb to γδ TCR or V regions within the gated population, was divided by percent of CD3+ T cells within the same gate. All mAb were purchased from T cell Sciences (Cambridge, Massachusetts, USA), and analyzed on an Epics profile II Coulter Electronic FACS as described previously [18]. MAb to CD4, CD8, and CD20 were from Becton Dickinson. A single analysis was performed for each patient. Differential CBC for enumeration of eosinophils were performed on an automated Coulter Counter.

### Statistics

Values in compared groups were normally distributed (Shapiro–Wilk test). One and two tailed Student's *T*-test to compare mean values and Pearson's correlation coefficient between groups of values were computed using Excel software. Means (*M*) and 1 SD were calculated and reported as M ± 1 SD.

## Results

### Patient and controls

Fifteen male and three consenting female patients, (mean age 26.8 ± 8.9 years), six with *S. mansoni*, 11 with *S. hematobium*, and one with mixed infection (Table1) were studied. None had previously been exposed to *Schistosoma*. All patients had evidence of a humoral immune response to schistosomal antigens at diagnosis, reflected by the presence of antibodies in the serum detected by ELISA and immunoblot. Eggs were recovered in urine or stool of six patients. Thirteen of the 18 patients had symptoms attributable to the disease (acute or chronic manifestations), whereas five were asymptomatic (Table1).

**Table 1:** Clinical data of infected patients

Patient (sex)	Symptoms	Clinical presentation	% γδ/CD3	Eosinophils total/mm^3^ (%)	Month after infection	Type	Type
1 (M)*	AS−		10^#^,19^##^,12^##^	4100 (31)	2	SM	SM
2 (M)	AS−		3″″	400 (6)	7	SM	SH
3 (M)*	AS−		1.4^#^, 2^##^	700 (8)	2	SM	SM
4 (M)	AS−		2^##^	1250 (18)	12	SM	
5 (M)	AS−	Developed cough after therapy	7^##^	2800 (26)	4	SM	
6 (M)	AS+	Katayama fever, cough	0^##^	1500 (17)	4	SM	
7 (M)	AS−	Fatigue	10^##^	90 (1.5)	36	SH	
8 (M)*	AS−	Hematuria and hematospermia	7^#^, 10^##^, 3^##^	750 (9)	3	SH	SH+
9 (M)*	AS−		6^#^, 16^##^	1330 (19)	3	SH	SH
10 (M)*	AS+	Cough and pulmonary infiltrate	2^#^, 3^##^	3700 (39)	3	SH	SH
11 (M)*	AS+	Cough + pul inf.	1.5^#^, 4.5^##^	680 (11)	3	SH	SH+
12 (F)*	AS+	Katayama fever + Pul infilt.	4^#^, 5.5^##^	930 (9)	3	SH	SH
13 (M)*	AS+	Pulmonary. Infiltrate and cough	3.8^#^, 4^##^	2900 (30)	3	SH	SH
14 (F)*	AS+	Katayama fever	1^#^, 1.3^##^	130 (2)	3	SH	SH
15 (F)*	AS+	Hematuria and h/o cough and pulmonary infiltrates	3.7^#^, 6^##^, 3	1800 (14)	8	SH	SH+
16 (M)	AS−	Hematuria and hematospermia	3	90 (1)	11	SH+	SH+
17 (M)	AS+	Katayama	1	4680 (36)	<2	SH/SM	SM + SH+
18 (M)	AS+	Katayama	2.3^#^, 2.7^##^	3020 (31)	<2	SH	SH

Asterix denotes patients studied both before and after course of treatment. # and ## denote respectively value before and after therapy. M, male; F, female, SM, infected with *S. mansoni*; SH, infected with *S hematobium*; +, eggs detected; AS, acute schistosomiasis syndrome; h/o, history of.

### γδ T cell response

**Figure 1:**
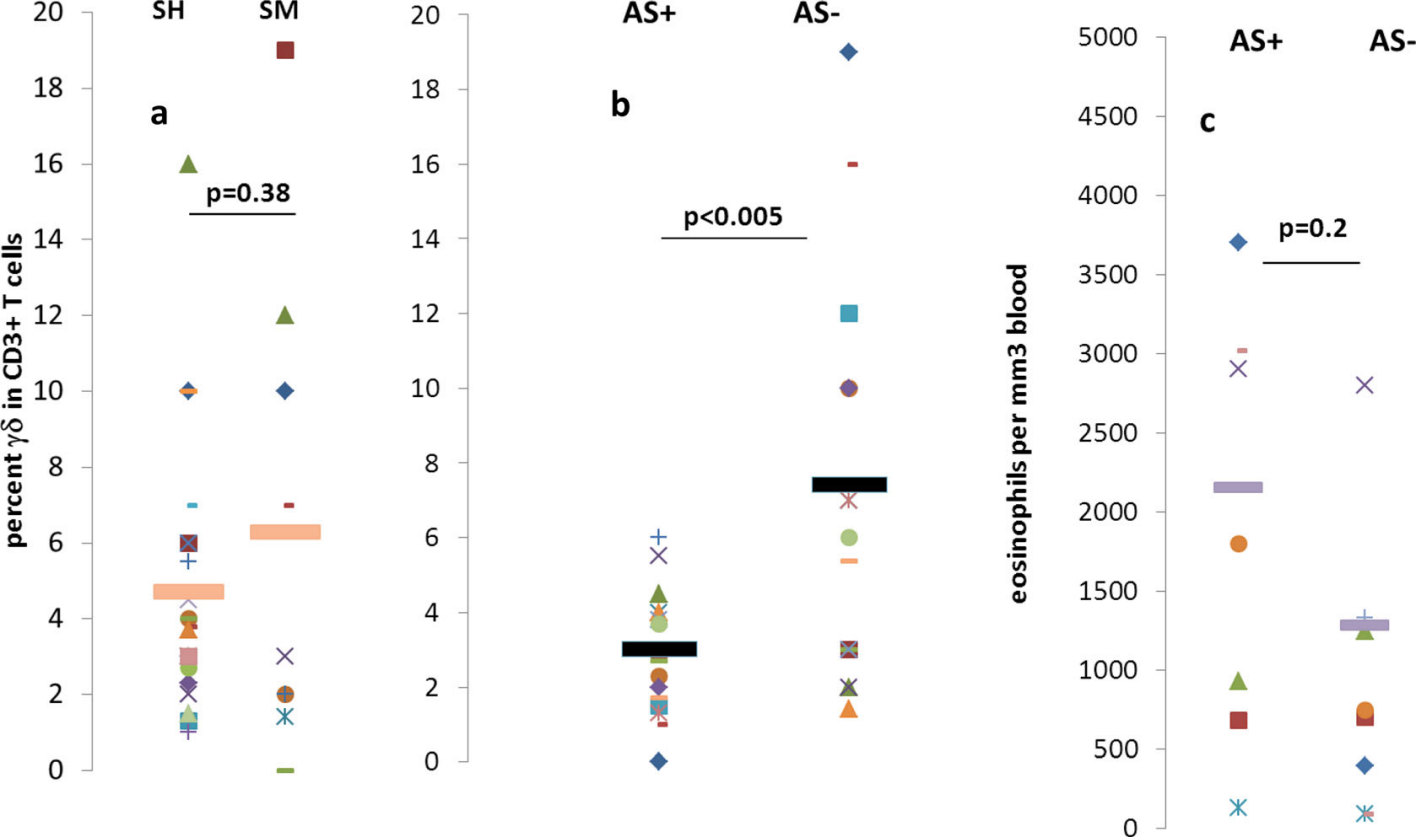
Correlation of γδ T cells with clinical presentation of schistosomiasis. γδ T cells (percent of all CD3+ T cells) (a and b) in the peripheral blood of patients with *Schistosoma mansoni* (SM, *n* = 9 evaluations) or *S. hematobium* (SH, *n* = 21 evaluations), and in patients with or without acute schistosomiasis (AS+, *n* = 15 and AS−, *n* = 15, respectively). Eosinophil counts per mm^3^ of blood are shown in (c). Means are denoted by horizontal bars and *P* values for comparison of means (Student's *T*-test) are indicated. ns = difference not significant.

The mean of the highest percentage of γδ T cells measured either before or after treatment among the 18 patients was 5.7 ± 2.1% of the PB CD3+ T cells compared to a control cohort of 16 healthy individuals (mean 3.7 ± 3.1%, *P* = 0.09) suggesting a trend toward an elevated level of γδ T cells in these patients during infection. There was no significant difference between the mean of the maximal percentage of γδ T cells among the total peripheral blood CD3+ T cells among *S. mansoni* and S*. hematobium* infected patients (4.1 ± 3.4% vs. 3.7 ± 1.8%, respectively) or in the mean for all evaluations performed in the respective patient groups (4.6 ± 3.5% vs. 6.2 ± 6.3%, *P* = ns, Fig. 1a). Patients with acute schistosomiasis (AS+) presented nonsignificantly earlier than those without (AS−) (3.2 ± 2.0 vs. 8.8 ± 10.8 months, *P* = 0.14), but there was no correlation between time of presentation when γδ T cells were evaluated, and maximal levels of γδ T cells in either AS+ or AS− patients (*R* = 0.49, *P* = not significant, *R* = −0.09, *P* = not significant, respectively).

**Figure 2:**
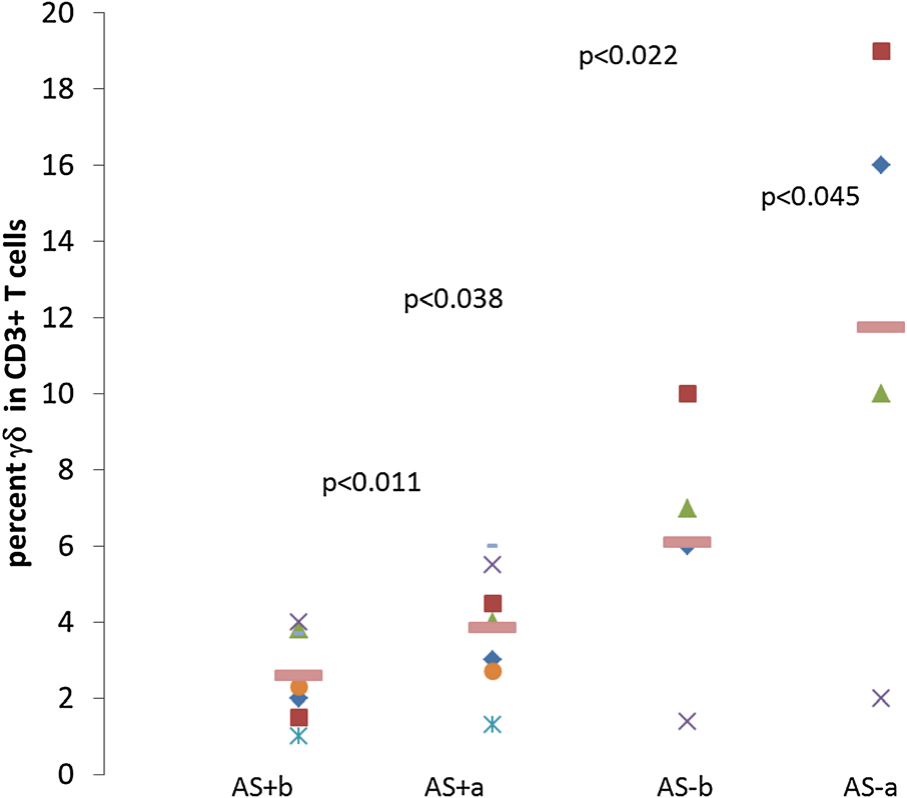
Effect of treatment on γδ T cells in schistosomiasis. γδ T cells as percent of peripheral blood CD3+ T cells in patients with or without acute schistomiasis (AS+, *n* = 6, AS−, *n* = 4, respectively), before (b) or after (a) treatment are shown. Mean values are denoted by horizontal bars and *P* values for comparison of means (Student's *T*-test) are indicated.

**Figure 3:**
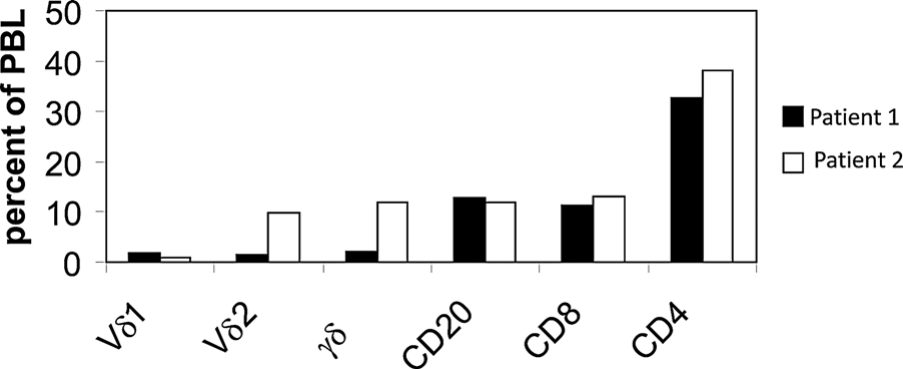
Lymphocyte subsets in two patients with schistosomiasis. Peripheral blood lymphocytes of two patients with schistosomiasis without a clinical presentation typical of acute schistosomal syndrome (AS−) were analyzed by FACS after staining with fluorescence labeled monoclonal antibodies to CD20, CD4, CD8, the Vδ2, Vδ1 gene segments and to a global marker of the γδ T cell receptor. Bars show the percentage of each subset among the peripheral blood lymphocytes (PBL).

Interestingly however, percent of γδ T cells among PB T cells of the nine AS+ patients (*n* = 15 evaluations) was significantly lower than in the nine AS− patients (*n* = 15 evaluations) (3.0 ± 1.7% vs. 7.4 ± 5.3%, respectively, *P* < 0.005) (Fig. 1b). Furthermore, comparison of the means of the highest percentages of γδ T cells among CD3+ T cells in the PB, measured in each of AS+ patients when one or more evaluations was performed, was also lower than in the AS− patients (3.2 ± 1.9% vs. 8.0 ± 6.2%, *P* < 0.045). Likewise, for evaluations done either before or after therapy, the percent γδ T cells was higher in the AS+ group (Fig. 2). In contrast to the significant differences in percent γδ T cells among the CD3+ T cells, and although the mean in AS+ patients was higher, the absolute number of eosinophils in the complete white blood cell counts of the two groups was not significantly different (2148 ± 1516 vs. 1278 ± 1345 *P* = 0.2) (Fig. 1c). In addition we found that, in both AS− and AS+ patients, γδ T cells expanded significantly after therapy (Fig. 2). Finally, to determine whether cells expanding in the patients bear characteristics consistent with a response to antigens produced in the mevalonate pathway, we stained the PBMC of two AS− patients, the first with elevated PB γδ TCR expressing cells (11.8% of lympocytes) and the second with no expansion of γδ T cells (2.2%) using mAb to the Vδ2 and Vδ1 gene products expressed in γδ TCR. As shown in Figure 3, in the patient with expanded γδ T cells, >90% expressed Vδ2 and only <10% expressed the Vδ1 gene segment. In contrast, among the T cells in the patient with no relative γδ T cell expansion there was a more even distribution of Vδ2+ and Vδ1+ lymphocytes. As shown in Figure 3, these patients had similar percentages of B cells, (CD20+), and of CD8+ and CD4+ T cells in the peripheral blood lymphocyte population. We also examined γδ T cells in relation to the development of pulmonary infiltrates (PI) in the patient cohort. Percent of γδ T cells among all peripheral blood T cells in patients with PI were lower than in patients without PI, although the differences were not statistically significant.

## Discussion

This is the first reported study, to our knowledge, of γδ T cells in non-immune patients with schistosomiasis, revealing differential responses in patients in accordance with the development or lack of the clinical syndrome of acute schistosomiasis. This syndrome occurs in a subset of non-immune patients exposed to *Schistosoma*-infected water and is also known as Katayama syndrome. Clinical manifestations include fever, cough, accompanied by pulmonary infiltrates, fatigue, arthralgias and myalgias, urticarial rash, angioedema, and abdominal pain [19]. The syndrome is unique to the non-immune population (usually travelers), appears 3–8 weeks after exposure to the *Schistosoma* cercaria, and may be related to immune complexes containing schistosomal antigens. In healthy individuals, the percent of γδ T cells within the total CD3+ T cell population is generally stable over time, and only change in specific stimulatory circumstances, for example, when patients are treated with intravenous administration of bisphosphonate for osteoporosis, which increases monocyte expression of IPP, a γδ T cell antigen, or in multiple sclerosis patients with active brain disease, by unknown mechanisms [20,21]. Our results now reveal for the first time, a relative expansion of γδ T cells within the total CD3+ T cell population in the PB in a group of patients from non-endemic areas who were infected with *Schistosoma*, following treatment of the infection. The drive for this expansion is currently unknown, but could be due to *Schistosoma* derived antigens. In this regard, our data, based on a patient in whom there was a large relative expansion of these cells, revealed that the majority of the relatively expanding γδ T cells expressed the Vδ2 gene. This finding, while based on limited data, is consistent with phosphorylated metabolites produced in the mevalonate pathway of the parasite, or, less likely, by host cells, being responsible for the relative expansion of γδ T cells among the CD3+ T cells in this patient with schistosomiasis. Genetic studies have indeed revealed expression of multiple enzymes of the mevalonate pathway in *Schistosoma*, including acetyl-CoA acetyltransferase [*S. japonicum* and *S. mansoni*] hydroxyl methyl glutaryl (HMG) – co-enzyme (Co) A reductase [*S. mansoni*], mevalonate kinase [*S. mansoni*] P-Mevalonate kinase [*S. japonicum*], mevalonate diphosphate decarboxylase [*S. mansoni*], and IPP isomerase in *S. japonicum* [22]. Thus, strong γδ T cell antigens such as IPP and its isomer, dimethylallyl pyrophosphate (DMAPP), are indeed produced by this parasite and could be responsible for γδ T cell activation and expansion within the peripheral blood T cell pool [23].

The differential increased relative expansion of γδ T cells in the PB in patients without, relative to patients with, the AS clinical syndrome even before therapy (Fig. 2) could be explained by host and parasite factors. For example, parasite loads and their metabolic activity, may differ between patients. For example, in acute *Mansoni* schistosomiasis studied in 26 Puerto Rican patients, severity of illness was found to be positively correlated (*r* = 0.79) with the intensity of infection as measured by the concentration of eggs of *S. mansoni* in stool specimens [24]. Compounded with our results indicating that in these patients γδ T cells would be less likely to expand, this suggests that in patients who develop the acute syndrome the ability of γδ T cells to proliferate in response to their cognate antigens is suppressed, perhaps due to the strong Th2 TCRαβ mediated response to the increased parasitic load. A similar low level of γδ T cells in patients exhibiting a potent Th2 like response is likewise found in allergic individuals, who have decreased γδ T cells in their PB [25]. It is possible, however, that in individuals manifesting clinically with AS, γδ T cells are distributed in the tissues where parasites localize, similar to their redistribution to the airways in allergic asthmatics [26].

On the other hand, the absence of systemic symptoms associated with a higher relative level of γδ T cells among the T cells in the peripheral blood, suggests that these cells may function to dampen the systemic acute inflammatory response engendered by the parasite, which is reminiscent of previous finding showing inverse relationship of γδ T cells and intensity of inflammation in juvenile idiopathic arthritis [27,28]. Recent studies suggest that γδ T cells could indeed suppress αβ T cells [29]. The observed increase of γδ T cells after therapy was instituted (Fig. 2), when the clinical syndrome is subsiding, together with data indicating that γδ T cells increase in the PB of malaria patients after therapy, lend additional support to this concept [18].

Further studies of the involvement of γδ T cells in schistosomal infections could lead to novel understanding of the host-parasite interactions and new therapeutic modalities. For example, bisphosphonates can upregulate IPP by blocking isopentenyl pyrophosphate synthase, thus boosting the γδ T cell response, which may have a beneficial clinical effect in patients with AS [15].

## References

[1] Gryseels B, Polman K, Clerinx J, Kestens L (2006). Human schistosomiasis. Lancet.

[2] Meltzer E, Artom G, Marva E, Assous MV, Rahav G, Schwartzt E (2006). Schistosomiasis among travelers: new aspects of an old disease. Emerg. Infect. Dis.

[3] Anthony RM, Rutitzky LI, Urban JF, Stadecker MJ, Gause WC (2007). Protective immune mechanisms in helminth infection. Nat. Rev. Immunol.

[4] Kaplan MH, Whitfield JR, Boros DL, Grusby MJ (1998). Th2 cells are required for the *Schistosoma mansoni* egg-induced granulomatous response. J. Immunol.

[5] Metwali A, Elliott D, Blum AM, Li J, Sandor M, Lynch R, Noben-Trauth N, Weinstock JV (1996). The granulomatous response in murine *Schistosomiasis mansoni* does not switch to Th1 in IL-4-deficient C57BL/6 mice. J. Immunol.

[6] Sabin EA, Kopf MA, Pearce EJ (1996). *Schistosoma mansoni* egg-induced early IL-4 production is dependent upon IL-5 and eosinophils. J. Exp. Med.

[7] McManus DP, Loukas A (2008). Current status of vaccines for schistosomiasis. Clin. Microbiol. Rev.

[8] Hayday AC (2000). [gamma][delta] cells: a right time and a right place for a conserved third way of protection. Annu. Rev. Immunol.

[9] Bank I, Marcu-Malina V Quantitative peripheral blood perturbations of γδ T cells in human disease and their clinical implications. Clin. Rev. Allergy Immunol.

[10] Eberl M, Hintz M, Reichenberg A, Kollas AK, Wiesner J, Jomaa H (2003). Microbial isoprenoid biosynthesis and human gammadelta T cell activation. FEBS Lett.

[11] Morita CT, Lee HK, Leslie DS, Tanaka Y, Bukowski JF, Marker-Hermann E (1999). Recognition of nonpeptide prenyl pyrophosphate antigens by human gammadelta T cells. Microbes Infect.

[12] Lindberg R, Johansen MV, Nilsson C, Nansen P (1999). An immunohistological study of phenotypic characteristics of cells of the inflammatory response in the intestine of *Schistosoma bovis*-infected goats. Parasitology.

[13] Sandor M, Sperling AI, Cook GA, Weinstock JV, Lynch RG, Bluestone JA (1995). Two waves of gamma delta T cells expressing different V delta genes are recruited into schistosome-induced liver granulomas. J. Immunol.

[14] Raziuddin S, Shetty S, Ibrahim A (1992). Phenotype, activation and lymphokine secretion by gamma/delta T lymphocytes from schistosomiasis and carcinoma of the urinary bladder. Eur. J. Immunol.

[15] Vandewaa EA, Mills G, Chen GZ, Foster LA, Bennett JL (1989). Physiological role of HMG-CoA reductase in regulating egg production by *Schistosoma mansoni*. Am. J. Physiol.

[16] Tsang VC, Wilkins PP (1991). Immunodiagnosis of schistosomiasis. Screen with FAST-ELISA and confirm with immunoblot. Clin. Lab. Med.

[17] Cetron MS, Chitsulo L, Sullivan JJ (1996). Schistosomiasis in Lake Malawi. Lancet.

[18] Schwartz E, Shapiro R, Shina S, Bank I (1996). Delayed expansion of V delta 2+ and V delta 1+ gamma delta T cells after acute *Plasmodium falciparum* and *Plasmodium vivax* malaria. J. Allergy Clin. Immunol.

[19] Leshem E, Maor Y, Meltzer E, Assous M, Schwartz E (2008). Acute schistosomiasis outbreak: clinical features and economic impact. Clin. Infect. Dis.

[20] Rinaldi L, Gallo P, Calabrese M, Ranzato F, Luise D, Colavito D, Motta M, Guglielmo A, Del Giudice E, Romualdi C (2006). Longitudinal analysis of immune cell phenotypes in early stage multiple sclerosis: distinctive patterns characterize MRI-active patients. Brain.

[21] Welton JL, Morgan MP, Marti S, Stone MD, Moser B, Sewell AK, Turton J, Eberl M (2003). Monocytes and gammadelta T cells control the acute-phase response to intravenous zoledronate: insights from a phase IV safety trial. J. Bone Miner. Res.

[22] Rajkovic A, Simonsen JN, Davis RE, Rottman FM (1989). Molecular cloning and sequence analysis of 3-hydroxy-3-methylglutaryl-coenzyme A reductase from the human parasite *Schistosoma mansoni*. Proc. Natl. Acad. Sci. USA.

[23] Venancio TM, DeMarco R, Almeida GT, Oliveira KC, Setubal JC, Verjovski-Almeida S (2007). Analysis of *Schistosoma mansoni* genes shared with Deuterostomia and with possible roles in host interactions. BMC Genomics.

[24] Hiatt RA, Sotomayor ZR, Sanchez G, Zambrana M, Knight WB (1979). Factors in the pathogenesis of acute *Schistosomiasis mansoni*. J. Infect. Dis.

[25] Chen KS, Miller KH, Hengehold D (1996). Diminution of T cells with gamma delta receptor in the peripheral blood of allergic asthmatic individuals. Clin. Exp. Allergy.

[26] Spinozzi F, Agea E, Bistoni O, Forenza N, Monaco A, Falini B, Bassotti G, De Benedictis F, Grignani F, Bertotto A (1995). Local expansion of allergen-specific CD30+ Th2-type gamma delta T cells in bronchial asthma. Mol. Med.

[27] Bendersky A, Marcu-Malina V, Berkun Y Cellular interactions of synovial fluid gammadelta T cells in juvenile idiopathic arthritis. J. Immunol.

[28] Berkun Y, Bendersky A, Gerstein M, Goldstein I, Padeh S, Bank I GammadeltaT cells in juvenile idiopathic arthritis: higher percentages of synovial Vdelta1+ and Vgamma9+ T cell subsets are associated with milder disease. J. Rheumatol.

[29] Peters C, Oberg HH, Kabelitz D, Wesch D Phenotype and regulation of immunosuppressive Vdelta2-expressing gammadelta T cells. Cell. Mol. Life Sci.

